# Mitochondrial non-energetic function and embryonic cardiac development

**DOI:** 10.3389/fcell.2024.1475603

**Published:** 2024-10-07

**Authors:** Jingxian Shi, Yuxi Jin, Sha Lin, Xing Li, Donghui Zhang, Jinlin Wu, Yan Qi, Yifei Li

**Affiliations:** ^1^ Key Laboratory of Birth Defects and Related Diseases of Women and Children of MOE, Department of Pediatrics, West China Second University Hospital, Sichuan University, Chengdu, Sichuan, China; ^2^ State Key Laboratory of Biocatalysis and Enzyme Engineering, School of Life Science, Hubei University, Wuhan, China

**Keywords:** mitochondria, cardiac development, embryo, non-energetic function, ROS

## Abstract

The initial contraction of the heart during the embryonic stage necessitates a substantial energy supply, predominantly derived from mitochondrial function. However, during embryonic heart development, mitochondria influence beyond energy supplementation. Increasing evidence suggests that mitochondrial permeability transition pore opening and closing, mitochondrial fusion and fission, mitophagy, reactive oxygen species production, apoptosis regulation, Ca^2+^ homeostasis, and cellular redox state also play critical roles in early cardiac development. Therefore, this review aims to describe the essential roles of mitochondrial non-energetic function embryonic cardiac development.

## Introduction

**FIGURE 1:**
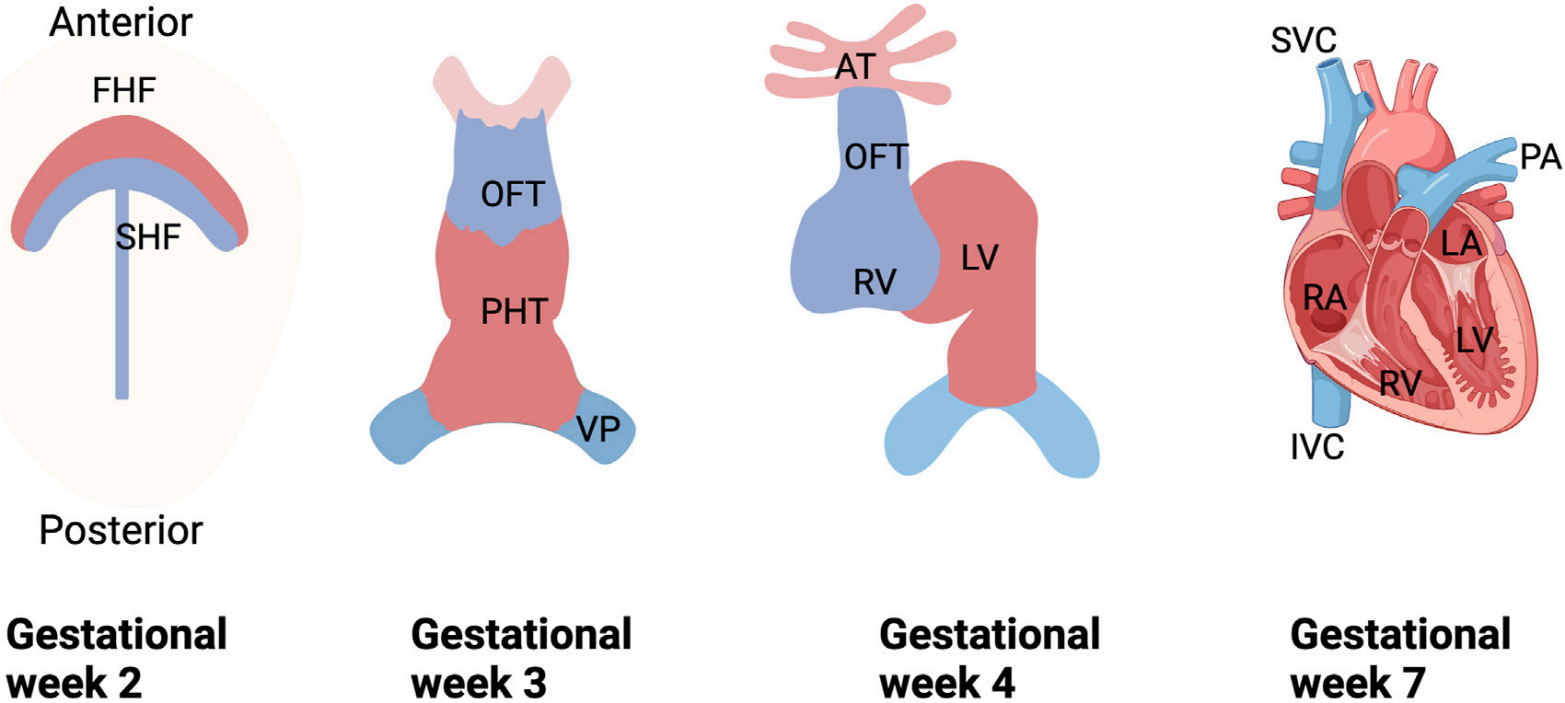
Schematic diagram of human embryonic heart development. During the 2nd week of gestation, cardiogenic mesodermal cells migrate towards the anterior side of the embryo to form FHF and SHF, which are designated to form specific regions of the PHT. During weeks 3 and 4 of gestation, FHF form the beating PHT and eventually give rise to the LV and portions of the right and left atria (RA and LA), and SHF are located posterior to the PHT and within the pharyngeal mesoderm to form the RV, portions of the atria and outflow tracts, and to form the base of the aorta and pulmonary arteries. And in the 3rd week of gestation, the cells of the venous pole form the superior and inferior vena cava (SVC and IVC), and ultimately, the four-chambered heart is formed by the end of the 7th week. FHF, first heart field; SHF, second heart field; PHT, primary heart tube; OFT, outflow tract; VP, venous pole; LV, left ventricle; RV, right ventricle; SVC, superior vena cava; IVC, inferior vena cava vein; PA, pulmonary artery.

During embryonic development in mammals, the heart is the first organ to develop and perform functionality (Bruneau, 2020; Buckingham et al., 2005). The primitive heart tube begins to beat for the first time on the twenty-second day of gestation (roughly equivalent to embryonic (E) day E8.5 in mice), allowing for active fetal circulation by the end of the fourth week of pregnancy (E9.5 in mice), culminating in the formation of a four-chamber heart by the seventh week of gestation (E13.5 in mice) (Tan and Lewandowski, 2020; Finnemore and Groves, 2015; Sánchez-Díaz et al., 2020). Cardiac development is a multi-step process that includes mesodermal differentiation, cardiac progenitor cell formation, and cardiac maturation (Zhu et al., 2020; Garry and Olson, 2006) (Figure 1). Recent studies have shown that the vast majority of cardiac cells are derived from Nkx2.5 cardiovascular progenitor cells (Stanley et al., 2002), the discovery of Nkx2.5 also ushered in a “golden age” of using molecular and genetic methods to study heart formation (Tanaka et al., 1999). Congenital heart defects resulting from abnormal heart development affect more than 1% of newborns (Wu et al., 2020). Accordingly, understanding and exploring the molecular and genetic mechanisms involved in the development of the heart embryo can provide important clues for better prevention and treatment of prevalent cardiac diseases.

Mitochondria, known as the power-factory of the cell, play a multifaceted role in the development and maturation of the embryonic heart (Gao and Zhang, 2018; Cheong et al., 2020). In almost all types of eukaryotic cells, mitochondria typically number in the hundreds within the cytoplasm of each cell, with each mitochondrion containing 2–10 copies of DNA (Harding, 1991; Andrieux et al., 2021). The specific number of mitochondria and their DNA copies generally correlate with the energy demands of the cell (Robin and Wong, 1988). As an organelle enveloped by a double phospholipid bilayer, the inner membrane of the mitochondrion encloses the matrix space and invaginates to form cristae (Vogel et al., 2006). This cristae structure maximizes the inner membrane’s surface area, providing ample attachment sites for various enzymes and electron transport chain complexes, thereby enhancing its ATP production capacity (Birkedal et al., 2022). The outer membrane, which encapsulates the inner membrane to form the intermembrane space, is highly permeable and creates a transitional space for the transport and storage of various metabolic substrates, facilitating enzymatic reactions occurring at the inner membrane (Gupta and Becker, 2021). As a semi-autonomous organelle, mitochondria contain approximately 1,500 proteins essential for their structure and function, with mitochondrial DNA (mtDNA) encoding less than 1% of these proteins, while the remainder are encoded by nuclear DNA (nDNA) (Nunnari and Suomalainen, 2012; Filograna et al., 2021; Vega et al., 2015). Unlike the long, linear structure of genomic DNA, mtDNA is a circular molecule consisting of about 16,000 base pairs (Chen Y. et al., 2014). Lacking introns, mtDNA has a high utilization rate and controls the expression of 37 genes, including 13 essential for the electron transport chain, 2 ribosomal RNAs, and 22 transfer RNAs (Chen Y. et al., 2014; Goffart et al., 2004; Bonekamp and Larsson, 2018; Becker et al., 2014). Mitochondria perform various cellular function, including ATP production, regulation of apoptosis, Ca^2+^ homeostasis, and their own homeostasis (Gao and Zhang, 2018; May-Panloup et al., 2021; Chandel, 2014; Shadel and Horvath, 2015; Gut and Verdin, 2013).

Nevertheless, there is accumulating evidence that mitochondria play a key role in the development of the embryonic heart, involving reactive oxygen species (ROS) production, cell apoptosis, mitochondrial fusion, and division. Accordingly, this paper focuses on the impact of non-energetic metabolism regarding mitochondria in embryonic cardiac development.

## The connection between mitochondria and the embryonic heart

Mitochondria not only produce more than 90% of the ATP required for cardiomyocyte function via oxidative phosphorylation (OXPHOS), but also play a pivotal role in biosynthesis, apoptosis, calcium homeostasis, cell signaling pathways, and genetic expression (May-Panloup et al., 2021; Chandel, 2014; Shadel and Horvath, 2015; Gut and Verdin, 2013).

The mitochondria in mammals are semiautonomous organelle composed of nuclear DNA (nDNA) and mitochondrial DNA (mtDNA) encoding proteins (Pagliarini et al., 2008; Gao et al., 2023). Although mtDNA is critical for OXPHOS, its role in maintaining cardiac function goes beyond energy supply. Hundreds of different pathogenic mitochondrial genome (mtDNA) mutations have already been identified in humans, many of which are associated with cardiac deficiencies (Bray and Ballinger, 2017). The mtDNA single-strand breaks and mtDNA-depleted cells are susceptible to apoptosis (Yen et al., 2005; Tann et al., 2011). The research showed that in mouse models missing mitochondrial components (such as those necessary for mtDNA maintenance or gene expression), embryonic development typically stalled at embryonic (E) day E8.5 (roughly equivalent to E20 in humans) and seemed to lack all cardiac structures (Cerritelli et al., 2003; Cámara et al., 2011). Mitochondrial protein encoded by the nucleus, mitochondrial transcription factor A (TFAM), is a protein required for the transcription of mitochondrial DNA (mtDNA) (Ngo et al., 2014). In a model of Nkx2.5Cre, the detection of TFAM inactivation leads to mitochondrial dysfunction, which induces ROS production to activate the DNA damage response, ultimately leading to embryonic lethal cardiac dysplasia (Zhang et al., 2018). Deletion of cardiomyocyte-specific mitochondrial phosphatase (PTPMT1) in the TnT-Cre mouse model leads to abnormal cardiac development and embryonic lethality between E16.5 and E18.5 (Chen et al., 2021; Zhang et al., 2011). Furthermore, mutations in mtDNA encoding OXPHOS subunit complexes IV and V were found in congenital heart disease patients (Heidari et al., 2022). Cardiovascular involvement occurs early in more than one-third of children with primary mitochondrial disease, with a mean age of onset of 5.7 ± 7.8 years, associated with a poor prognosis (Brambilla et al., 2020; Holmgren et al., 2003).

During cardiac development, the maturation of mitochondrial structure and function is accompanied by an increase in mitochondrial number and the formation of lamellar cristae (David et al., 1981; Dorn et al., 2015). *In vitro*, early cardiomyocyte differentiation, mitochondria are relatively sparse, rounded, and dispersed throughout the cytoplasm, with increasing numbers of mitochondria as the development (Lyra-Leite et al., 2021; Kim et al., 1992). In the fetal mouse heart, mitochondria appear as elongated tubular organelles at embryonic (E) day E9.5, which are ovoid containing more endomembrane invaginations (cristae) at E13.5 (Maroli and Braun, 2021). The mitochondria permeability transition pores (mPTP) open at E9.5, but close mPTP at E13.5 (Murray et al., 2014). Moreover, mitochondria in fetal cardiomyocytes are fewer in number and more confined to the center of the cell, and energy metabolism in fetal myocardium is less dependent on oxidative phosphorylation and correlates with the mechanisms by which fetal myocardium resists hypoxia (Agata et al., 2019). All of these findings demonstrate that mitochondria are closely related to embryonic heart development.

## Mitochondrial energy metabolism and embryonic heart development

**FIGURE 2:**
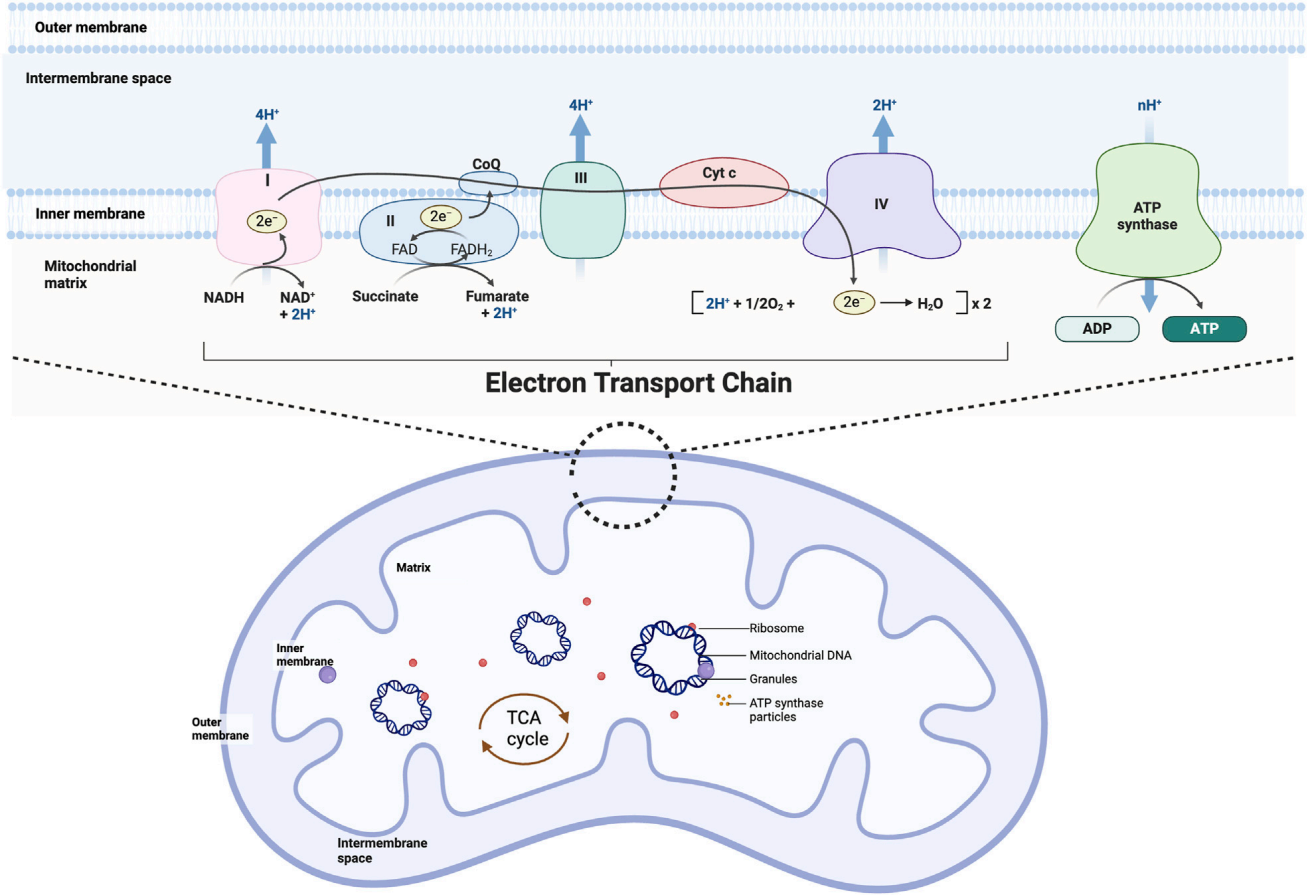
Mitochondrial structure and oxidative phosphorylation. Mitochondria can be divided from the outside to the inside into four functional regions: the outer mitochondrial membrane, the mitochondrial intermembrane space, the inner mitochondrial membrane, and the mitochondrial matrix, which generally also contains the mitochondria’s own DNA (mitochondrial DNA), RNA, and ribosomes (mitochondrial ribosomes). Mitochondrial oxidative phosphorylation is the movement of electrons through a complex (I-V) embedded in the inner mitochondrial membrane generates an electrical gradient through which protons are simultaneously pumped into the intermembrane space, producing a proton gradient. Together, the electrical gradient and the proton gradient generate electrochemical forces that lead to the synthesis of ATP by ATP-synthase.

Cardiac differentiation started in an early stage of embryonic progression (Kirby, 2002). During early cardiac development, energy metabolism transitions from reliance on anaerobic glycolysis to aerobic oxidative phosphorylation (Chung et al., 2007; Lopaschuk and Jaswal, 2010; Zhao et al., 2022) (Figure 2).

Previous studies have shown that at the early stage of mouse heart development (E9.5), energy is primarily derived from glycolysis, and the activities of mitochondrial electron transport chain (ETC) complexes are at low levels (Tanimura and Shepard, 1970; Lai et al., 2008). Further measurements of oxygen concentration in the heart and other embryonic tissues indicate that oxygen concentration in cardiac tissue is only half that of other embryonic tissues, corroborating the low level of aerobic metabolism at this early stage of heart development (Burton, 2009). After E9.5, researchers observed a significant increase in the proportion of aerobic metabolism and mitochondrial ATP production, while glucose metabolism levels declined. As heart development progresses, from E8.5 to E14 in mice, the activities of, ETC complexes I, II, III, and V markedly increase, and mitochondria in the heart begin to actively participate in oxidative phosphorylation (Ingraham et al., 2009). Postnatally, the activity and quantity of ETC complexes continue to rise, with reliance on glycolysis and lactate metabolism decreasing until the development of mature cardiomyocytes, where fatty acid oxidation provides 90% of the energy (Lopaschuk and Jaswal, 2010; Zhao et al., 2022). These studies clearly demonstrate that the proportion of mitochondrial metabolism gradually increases during the development and maturation of the heart.

## Mitochondrial non-energetic and embryonic heart development

**FIGURE 3:**
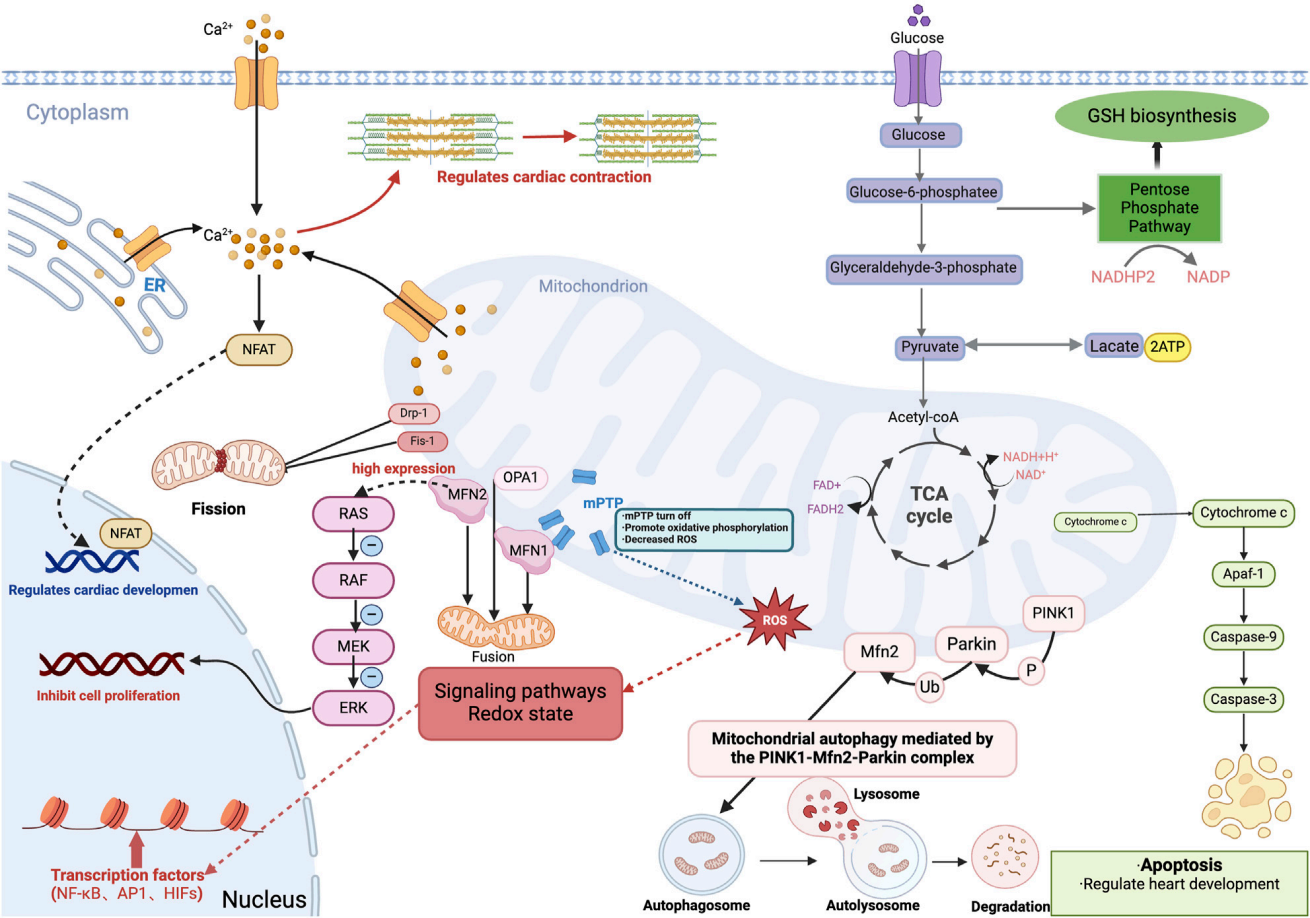
Role of mitochondria in embryonic heart development. Cardiomyocytes produce energy (ATP) by oxidative phosphorylation or anaerobic glycolysis, and during embryonic heart development, energy metabolism shifts from anaerobic glycolysis to aerobic oxidative phosphorylation involving mitochondria. And mitochondria produce intermediary metabolites and reactive oxygen species (ROS), which are involved in signaling pathways, redox homeostasis, and gene expression, and involve transcription factors including the κB family (NF-κB), activator protein-1 family (AP1) and hypoxia-inducible transcription factors (HIFs). Additionally, mitochondrial release of cytochrome c mediates apoptosis and regulates cardiac development during the embryonic period. Also, the closure of the mitochondrial permeability transition pore (mPTP) during the embryonic period facilitates oxidative phosphorylation, which reduces ROS levels in embryonic cardiomyocytes and promotes differentiation of embryonic cardiomyocytes. Mitochondrial quality control, including mitochondrial fusion, division, and mitochondrial autophagy is mediated by the PINK1-Mfn2-Parkin complex; Mitochondrial protein-2 (MFN2) regulates mitochondrial morphology and function by participating in mitochondrial fusion. While MFN2, which is highly expressed in embryonic stage, inhibits cardiomyocyte proliferation through the Ras-Raf-ERK signaling pathway. Mitochondria in the embryonic heart binds to ER and mediates the regulation of cardiac systolic-diastolic processes by Ca^2+^ concentration, and also activates the Ca ^+^-dependent NFAT signaling pathway to regulate cardiac development. GSH, glutathione. ER, endoplasmic reticulum.

The heart requires a constant supply of ATP from mitochondria for contraction and relaxation, but damaged mitochondria become a source of reactive ROS and cell death signals under pathological conditions (Saito et al., 2021; Saito and Sadoshima, 2015). More and more evidence suggests that mitochondria can influence cardiomyocyte differentiation and progression through more subtle means (Pohjoismäki and Goffart, 2017). For example, the regulation of reactive oxygen species (ROS) production and apoptosis, the opening and closing of the mitochondrial permeability transition pore, the dynamic balance between mitochondrial fusion and fission, Ca^2+^ homeostasis, and the regulation of the cellular redox state should not be neglected (Figure 3).

## Pros and cons of embryonic ROS production

Most of the intracellular reactive oxygen species (ROS) are generated by mitochondrial metabolism (Birket et al., 2013). During electron transfer in mitochondria, premature oxidation of some electrons occurs, resulting in the production of ROS (Semenza, 2007; Zorov et al., 2014). The major ROS molecules in the body include superoxide anion (O_2_-), hydroxyl radical (•OH), and hydrogen peroxide (H_2_O_2_) (Shao et al., 2012). Initially, ROS, a byproduct of the electron transport chain, was traditionally thought to primarily damage DNA and cause strand breaks (Dennery, 2010). However, contemporary research increasingly suggests that ROS can regulate various biological processes (Sauer and Wartenberg, 2005). In the early development of the heart, ROS acts as a signaling molecule playing a crucial role (Thompson and Al-Hasan, 2012; Sauer et al., 1999).

There may be a delicate balance between oxygen levels and the production of ROS during the gestation period of embryonic growth. On the one hand, ROS can promote the differentiation efficiency of cardiomyocytes through multiple signaling pathways such as JNK, ERK1/2, p38, Ca^2+^, BMP, and increase the percentage of beating cardiomyocytes in embryoid bodies (Buggisch et al., 2007; Wei and Cong, 2018). High levels of ROS in embryonic stem cells are associated with an increase in the percentage of beating cardiomyocytes in embryos within the body (Sauer and Wartenberg, 2005). Moreover, hypoxia in the fetal rat heart induces epigenetic repression of the protein kinase C epsilon (PKCɛ) gene through ROS-mediated pathways, resulting in cardiac susceptibility to ischemic injury in the fetal heart (Hellgren et al., 2021; Patterson et al., 2012). While N-acetylcysteine as a ROS scavenger inhibits hypoxia-induced methylation of the SP1 binding site, decreases cardiac susceptibility to ischemic injury by increasing SP1 binding to the PKCɛ promoter (Patterson et al., 2012). As a result, ROS can enhance the differentiation of embryonic stem cells to cardiac cells.

On the other hand, high levels of ROS due to mitochondrial dysfunction may lead to severe congenital heart disease. High ROS levels lead to DNA damage and initiate a DNA damage response pathway that causes cardiomyocyte cell cycle arrest during embryonic development (Puente et al., 2014). The nucleus-encoded mitochondrial protein, TFAM (mitochondrial transcription factor A), is required for mtDNA transcription (Ngo et al., 2014). In the Nkx2.5-Cre model, TFAM deactivation leads to mitochondrial dysfunction, which induces ROS production to initiate a DNA damage response, ultimately contributing to embryonic lethal myocardial dysplasia (Zhang et al., 2018). The anticonvulsant drug valproic acid raises intracellular ROS levels in mouse embryoid somata, which can suppress cardiomyocyte polarization and lead to cardiac malformations (Na et al., 2003). The mitochondria-targeting antioxidant Mitoquinone (MitoQ), which consists of lipophilic triphenylphosphine (TPP) cations covalently attached to the antioxidant ubiquinone, prevents superoxide-induced lipid peroxidation and mitochondrial damage (Smith et al., 2008; Smith and Murphy, 2010). In chronically hypoxic chicken embryos, MitoQ treatment restores the mitochondrial respiratory control ratio, left ventricular structure, and contractile function of the heart by suppressing mitochondrial derived oxidative stress in embryonic heart (Botting et al., 2020). ROS undergo complex movements during embryonic cardiac development, whereby early ROS levels are increased to stimulate cardiomyocyte differentiation, whereas at later stages, ROS levels are reduced to allow cardiomyocyte maturation by shutting down mPTP (Hom et al., 2011).

## Mitochondria participate in apoptosis

Mitochondria play a crucial role in regulating programmed cell death (apoptosis) (Jeong and Seol, 2008; Zhao et al., 2019; Fuchs and Steller, 2011). Mitochondria can mediate apoptosis by releasing cytochrome c (Cyt c), which binds to procaspase-9 and Apaf-1, subsequently activating procaspase-3 and ICAD downstream, leading to uncontrolled cleavage of nuclear DNA and initiating apoptosis (Green and Reed, 1998; Li et al., 2000; Lakhani et al., 2006). Studies have shown that mice lacking Apaf-1 typically exhibit severe developmental defects, and mutation at position 72 of cytochrome c results in similar phenotypes, underscoring the regulation of mitochondrial apoptotic pathways by cytochrome c (Nagasaka et al., 2010). Apoptosis also regulates multiple processes in cardiac development (Schaefer et al., 2004; Sharma et al., 2004; Maguire et al., 2017). Watanabe initially reported that cardiac outflow tract (OFT) shortening and rotation in chicken embryos occur through apoptosis of myocardial cells in the proximal OFT region (Watanabe et al., 1998). Studies in mouse embryonic hearts have confirmed the universality of this phenomenon, observing apoptosis initiation in the proximal OFT region around E12.5, peaking between E13.5 and E14.5, followed by a decline as development progresses (Barbosky et al., 2006). In addition to the OFT region, apoptosis has been detected in ventricular and endocardial cells during development, suggesting a potential role of apoptosis in ventricular morphogenesis (Watanabe et al., 2001).

## Mitochondrial permeability transition pore opening and closing in cardiac development

The mitochondrial permeability transition pore (mPTP) is an electrical conduction channel in the inner mitochondrial membrane (Giorgio et al., 2018). Under physiological conditions, mPTP is turned off in mature cardiomyocytes (Beutner et al., 2017). Previous studies have demonstrated that the mPTP channel persists in mouse embryos from E9.5 to E13.5. Closure of the mPTP enhances mitochondrial membrane potential (Δψ) and facilitates OXPHOS, leading to decreased ROS levels in embryonic cardiomyocytes. These findings suggest that mPTP closure acts upstream in redox signaling, thereby stimulating cardiac maturation (Hom et al., 2011). In addition, it was found that heterogeneity in the degree of cardiomyocyte differentiation within the embryonic heart may then be associated with differential regulation of mPTP activity in the ventricular wall (Drenckhahn, 2011). Furthermore, to inhibit the mouse adenine nucleotide transporter 2 (ANT2) gene showed impaired cardiac development and depressed maturation of embryonic cardiomyocytes, which may be mainly related to altered regulation of mitochondrial mPTP (Kokoszka et al., 2016; Levy et al., 2000). *In vitro*, using cyclosporin A (CsA) promotes cardiomyocyte differentiation from pluripotent stem cells (PSCs) by inhibiting mPTP and activating mitochondrial oxidative metabolism (Cho et al., 2014). All these indicate that mPTP may be involved in regulating embryonic heart development and enhancing cardiomyocyte differentiation.

## Fusion and fission of mitochondria in embryonic heart development

Mitochondria are a highly dynamic network system that is constantly remodeling mitochondria and maintaining their homeostasis through the processes of fusion and fission (Ma et al., 2020). Fusion is mediated by mitofusins-1 and mitofusins-2 (MFN1 and MFN2) on the outer membrane together with the inner membrane protein optic atrophy 1 (OPA1) (Adebayo et al., 2021). Fission is controlled by dynamin-related protein 1 (Drp1) and fission protein 1 (Fis1) (Wang et al., 2020). Both maintain dynamic balance, which is essential for the maintenance of normal mitochondrial morphology, distribution, and function (Mu et al., 2020; Shih and Robinson, 2018).

During the development and maturation of cardiomyocytes, there are significant changes in the number and size of mitochondria. To uncover the potential functions of mitochondrial fusion and fission in the process of cardiac development. Researchers utilized Nkx2.5-Cre to perform simultaneous deletion of Mfn1 and Mfn2 in embryonic hearts, resulting in embryonic lethality in experimental mice (Moses et al., 2001; Chen et al., 2011; Chen et al., 2003). Subsequent studies in embryonic stem cells demonstrated that blocking mitochondrial fusion diminishes the capacity of Ca^2+^ uptake into mitochondria and increases cytoplasmic Ca^2+^ concentration, leading to cellular demise (Kasahara et al., 2013). Conversely, employing Myh6-Cre to concurrently deactivate Mfn1 and Mfn2 in embryonic hearts from E15.5 to P0 showed no immediate cardiac dysfunction; however, these mice developed dilated cardiomyopathy starting 7 days after birth and all perished before reaching P16 (Papanicolaou et al., 2012). Another recent study also highlighted unbalanced mitochondrial dynamics and mitochondrial bioenergetics in bicuspid aortic valve (BAV) tissues together with reduced expression of Notch1 (Abudupataer et al., 2021). All these evidences support the important role of mitochondrial fusion in the developmental differentiation of cardiomyocytes (Tahrir et al., 2019; Dorn, 2016b; Stanley et al., 2005; Gong et al., 2015; Dorn, 2016a).

Besides, mutations in desmin during early embryonic development led to a significant increase in Drp1, subsequently causing notable cardiac dysfunction (Goldfarb and Dalakas, 2009; Alam et al., 2018). Further investigations using Myh6-Cre in mice to delete the mitochondrial fission protein Drp1 have demonstrated multiple cardiac defects, including reduced heart rate, abnormal electrocardiographic patterns, and decreased left ventricular contraction (Kageyama et al., 2014). All mutant mice succumbed between P9 and P11 (Kageyama et al., 2014). Specific deletion of Drp1 in mouse hearts at 4 weeks postnatally results in cardiac hypertrophy and death within 2 weeks (Ikeda et al., 2015). Conversely, deletion at 8 weeks reveals significant left ventricular enlargement and decreased ejection function, culminating in widespread heart failure 6–7 weeks post-deletion (Song et al., 2017; Song et al., 2015). These results underscore the essential role of mitochondrial fission in early cardiac development, with diminishing demand as maturity progresses (Dorn, 2015).

Furthermore, studies have shown that in addition to the involvement of MAFN2 in mitochondrial fusion to regulate mitochondrial morphology and function (de Brito and Scorrano, 2009; Santel, 2006), this gene has an important role in controlling cell proliferation and apoptosis (Guo et al., 2007). Maternal obesity and diabetes can increase the risk of cardiac disease in developing infants (Brite et al., 2014; Pauliks, 2015; Basu and Garg, 2018). In an animal study in rats, prenatal exposure to a high-fat diet led to an expression increase of the MFN2 protein, which affected mitochondrial viability in the developing fetus; moreover, the high expression of MFN2 depressed cell proliferation through the Ras-Raf-ERK signaling pathway, High expression of MFN2 during cardiac development may contribute to a decrease in the number of cardiomyocytes, secondary hypertrophy, as well as an impairment of diastolic and systolic functions (Chen K. H. et al., 2014; Chandhok et al., 2018; Larsen et al., 2019).

## Mitophagy in cardiac development

Mitochondria as the powerhouse play a key role in the cell. As a defense mechanism, mitophagy selectively removes damaged and dysfunctional mitochondria from the cell in order to maintain mitochondrial mass and thus maintain mitochondrial physiological function (Campos et al., 2016). In mouse fetal cardiomyocytes, mitochondria were found to undergo mitochondrial autophagy mediated by PINK1-Mfn2-Parkin complexes (Tahrir et al., 2019). Through the phagocytic mechanism of PINK1-Mfn2-Parkin, the mitochondrial transition from fetal carbohydrate metabolism to normal fatty acid metabolism in postnatal life can be facilitated (Dorn, 2016b). In addition, in mouse embryonic cardiomyocytes, Mfn2 mutation leads to deletion of the PINK1 phosphorylation binding site, which inhibits mitochondrial maturation and leads to metabolic arrest, ultimately causing fatal cardiomyopathy (Gong et al., 2015; Dorn, 2016a). Accordingly, Parkin-mediated mitochondrial autophagy is essential for the maturation of normal perinatal cardiac mitochondrial metabolism.

## Ca^2+^ homeostasis

Mitochondria possess several Ca^2+^ transport channels, including the Ca^2+^ uniporter complex, the mitochondrial Na^+^/Ca^2+^ exchanger, and the mitochondrial H^+^/Ca^2+^ exchanger, which, in conjunction with the endoplasmic reticulum, regulate the stability of mitochondrial Ca^2+^ levels (Kuster et al., 2010; Song et al., 2018; Berridge et al., 2000). Ca^2+^ plays a crucial role in cardiac development and maturation, acting as a second messenger involved in nearly all cellular biological processes during early development (Berridge et al., 2003; Créton et al., 1998). The critical role of increased Ca^2+^ concentration during embryonic development was first recognized during fertilization (Ducibella et al., 2006). Furthermore, mitochondria in the embryonic heart regulate cardiac development by activating the Ca^2+^-dependent NFAT signaling pathway (Guo et al., 2002; Cao and Chen, 2009). In cardiomyocytes, during cardiac contraction, intracellular Ca^2+^ accumulates, promoting the interaction between actin and myosin. During cardiac relaxation, intracellular Ca^2+^ decreases, leading to the decoupling of myofilaments and subsequent relaxation of cardiomyocytes (Bers and Despa, 2006; Aronsen et al., 2013). Therefore, mitochondria are essential for maintaining intracellular Ca^2+^ stability, which is vital for cardiac development and maturation.

## Mitochondria regulate cellular redox state

The ROS levels are tightly regulated by the antioxidant system under physiological conditions (D’Autreaux and Toledano, 2007), In order to protect mitochondria from ROS, the GSH (γ-L-glutamyl-L-cysteinyl-glycine) pool of antioxidants is essential for the protection of mitochondrial function and cell survival (Quintana-Cabrera and Bolaños, 2013; Fahey et al., 1984). Studies have found that GPx-4 (glutathione peroxidase 4) deficient embryonic mice die at E7.5 (Yant et al., 2003). Prxs (peroxiredoxins) are a family of proteins that are very effective in scavenging peroxides (Cox et al., 2009). Whereas Prx III is an important H_2_O_2_-eliminating enzyme in mitochondria, intracellular ROS levels were significantly higher in macrophages from Prx III knockout mice, and Prx III depletion induced apoptosis susceptibility (Chang et al., 2004; Li et al., 2007).

In addition, mitochondria maintain cytosolic NAD^+^/NADH redox homeostasis through malate-aspartate shuttling and glycerophosphate shuttling activities (Broeks et al., 2021; Xiao et al., 2018). NAD^+^ adjusts the acetylation of mitochondrial proteins by acting as a coenzyme for protein deacetylases such as sirtuins (Chang and Guarente, 2014). Its deficiency decreases sirtuins activity, causing a decrease in placental function and resulting in increased oxidative stress, inflammation, altered placental metabolism, as well as decreased trophoblast viability (Kahmini et al., 2022; Teixeira et al., 2020; Alqarni et al., 2021). Complex I deficiency causes NADH accumulation and reduced NAD^+^/NADH ratio, depresses SIRT3 activity, resulting in hyperacetylation of mitochondrial proteins in cardiac tissues (Karamanlidis et al., 2013). Whereas mouse SIRT1 knocks die during fetal development or shortly after birth as well as exhibit severe developmental defects, including heart defects (Rodriguez et al., 2013). Consequently, the role of mitochondrial mechanisms in maintaining NAD^+^/NADH redox homeostasis in coordinating cardiac development cannot be ignored (Hocaoglu and Sieber, 2023).

## Conclusion

Accumulating evidence indicates that mitochondria are not only the powerhouses driving the heart’s function, but also play a critical role in regulating mammalian embryonic heart development through non-energetic metabolic processes. This review highlights the regulatory roles of mitochondrial non-energetic metabolism in embryonic heart development, including the structure of the mPTP of mitochondria, fusion, fission and mitochondrial autophagy to maintain its homeostasis, as well as the regulation of ROS production, apoptosis, Ca^2+,^ and cellular redox state, all of which reveal the roles of mitochondria and the potential mechanisms in embryonic heart development, aiming at the early detection, treatment of mitochondrial dysfunction-induced heart defects in the embryonic stage, to provide clues for the development of new clinical applications.
